# Supplementary material for: Multiplex generation and single cell analysis of structural variants in a mammalian genome
Source: bioRxiv. 2024 Feb 12:2024.01.22.576756. Preprint. [Version 2] doi: 10.1101/2024.01.22.576756 (PMC10888807; doi:10.1101/2024.01.22.576756)
Supplement: 1 [file NIHPP2024.01.22.576756v2-supplement-1.pdf]

bioRxiv preprint doi: https://doi.org/10.1101/2024.01.22.576756; this version posted February 12, 2024. The copyright holder for this preprint (which was not certified by peer review) is the author/funder, who has granted bioRxiv a license to display the preprint in perpetuity. It is made available under aCC-BY 4.0 International license.

Supplementary Figures/Movie

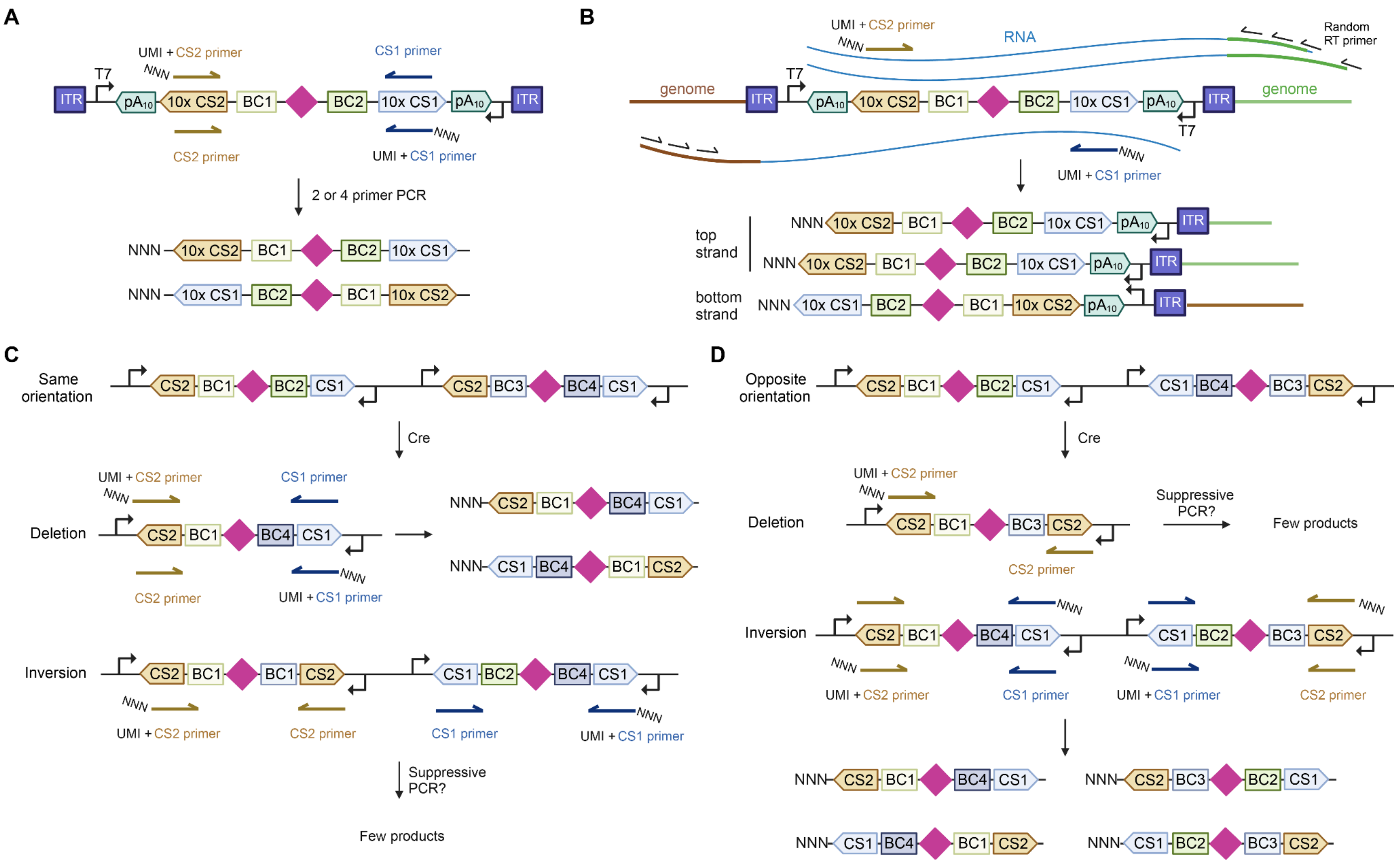

Fig S1. Schematic of sequencing library construction strategies for amplicon-seq and IVT-seq. A) Schematic of the shuffle cassette bearing the loxPsym site (diamond) flanked by two barcodes (BC1, BC2). Two 10x Genomics capture sequences (CS1, CS2) serve as binding sites for PCR primers for sequencing library construction. Only amplicons generated using one unique molecular identifier (UMI)-containing primer and one non-UMI primer can cluster and be successfully sequenced on an Illumina flow cell due to the sequencing adapters they encode. **B)** After IVT, transcripts are generated from both the top and bottom strand T7 promoters, and are expected to contain both BC1 and BC2 as well as adjacent genomic sequences from one side of the integrated shuffle cassette. Reverse transcription (RT) is performed with a primer containing 8 random bases at its 3' end. PCR is performed with a UMI-containing primer and one primer annealing to the constant sequence in the RT primer to yield the final sequencing library. **C)** and **D)** Recombination between two insertions *in cis* can lead to an inversion or deletion with shuffle cassettes containing either the same or different capture sequences. Theoretically, PCR products from a cassette with the same CS should amplify and cluster on an Illumina flowcell when libraries are generated using all 4 primers. However, empirically we find that these products are not readily detected, probably due to suppressive PCR (*52*, *53*) (see **Fig. S7B**).

bioRxiv preprint doi: https://doi.org/10.1101/2024.01.22.576756; this version posted February 12, 2024. The copyright holder for this preprint (which was not certified by peer review) is the author/funder, who has granted bioRxiv a license to display the preprint in perpetuity. It is made available under aCC-BY 4.0 International license.

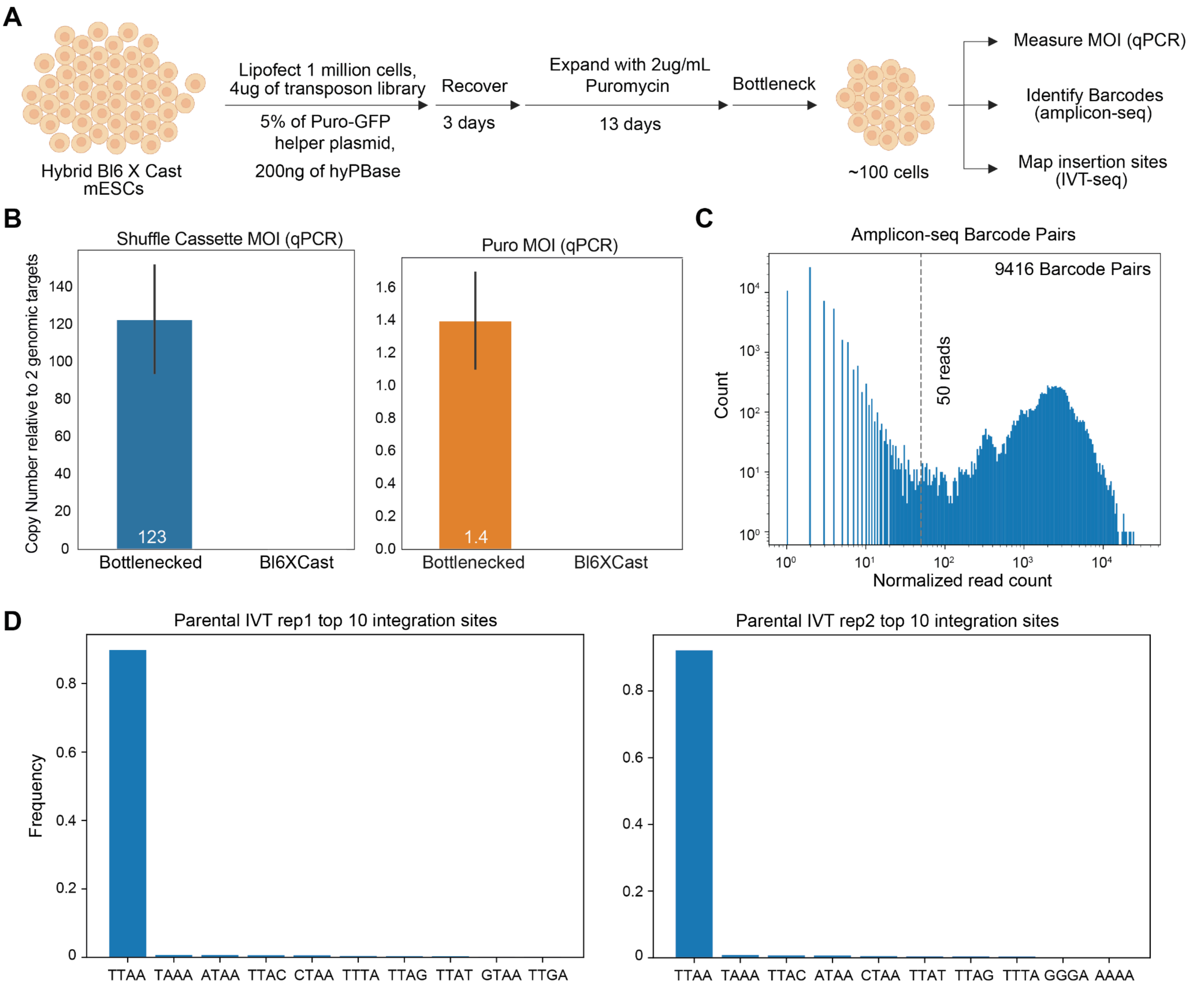

Fig S2. Integration and characterization of shuffle cassette library into mESCs. A) Schematic of experiment to integrate shuffle cassettes to the genomes of mESCs at a high multiplicity of infection (MOI) by co-transfection with a small percentage of a helper plasmid containing the puromycin resistance gene (*40*). **B)** Copy number of shuffle cassettes and the puromycin resistance gene were estimated in the bottlenecked population via quantitative PCR (qPCR) relative to two genomic targets (Trfc, Tert). The height of the bar represents the mean and the error bars indicate the standard deviation of the copy number measured relative to the two genomic targets . **C)** Histogram of read count for each barcode pair detected in amplicon-seq data normalized to sequencing depth across 4 technical replicates. **D)** Frequency of the first 4 bp of the genomic sequence detected in IVT-seq reads in technical replicate 1 and 2 from parental cells. TTAA is the expected sequence given our use of the PiggyBac transposon.

bioRxiv preprint doi: https://doi.org/10.1101/2024.01.22.576756; this version posted February 12, 2024. The copyright holder for this preprint (which was not certified by peer review) is the author/funder, who has granted bioRxiv a license to display the preprint in perpetuity. It is made available under aCC-BY 4.0 International license.

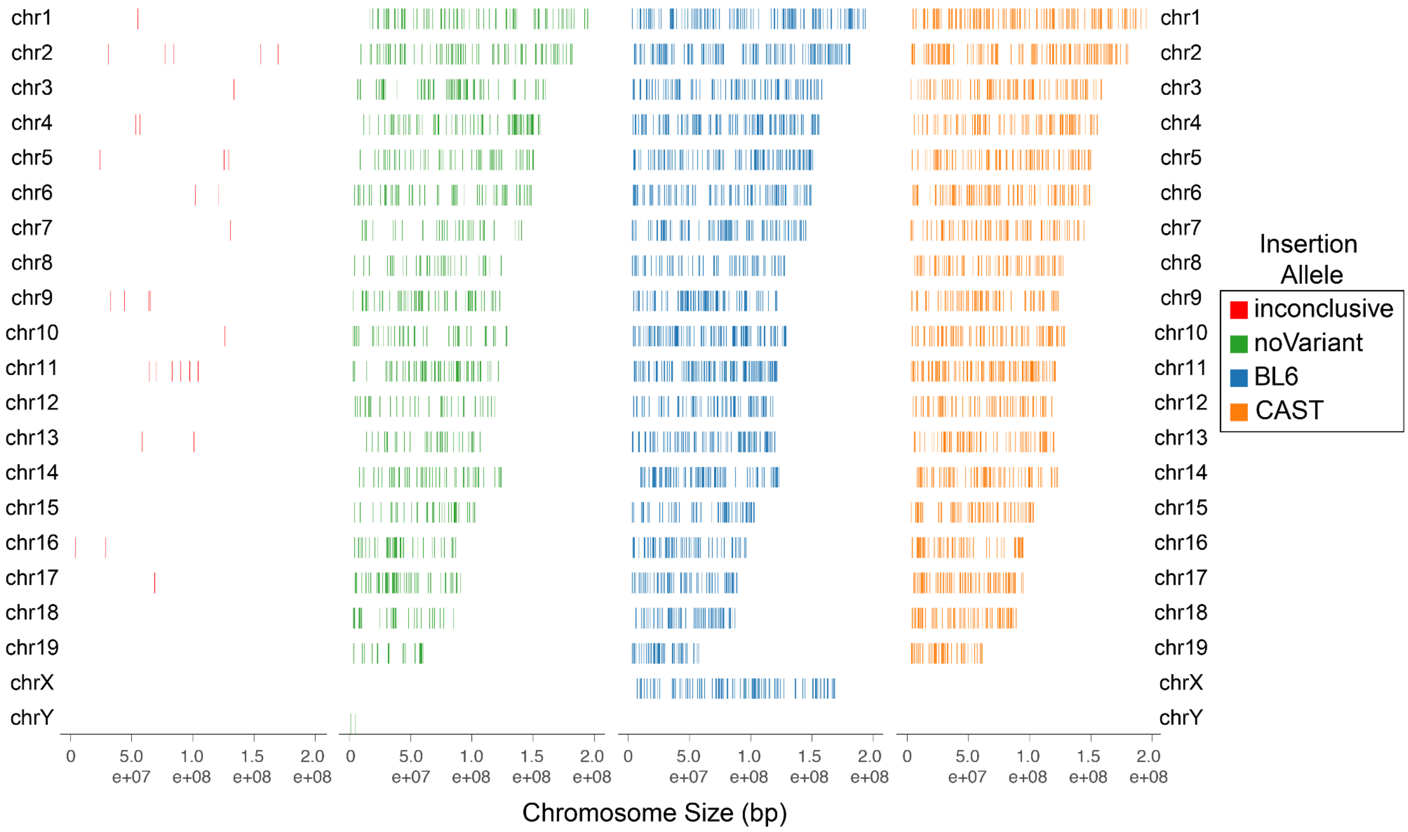

Fig S3. Allele-specific insertion sites across all chromosomes. Insertion sites across all chromosomes for shuffle cassettes whose genomic coordinates were mapped with high confidence, colored by allele. Inconclusive indicates that there is conflicting evidence for the insertion allele, while noVariant denotes those insertions that were un-assigned due to a lack of reads that overlap with a known variant between the BL6 and CAST genomes.

bioRxiv preprint doi: https://doi.org/10.1101/2024.01.22.576756; this version posted February 12, 2024. The copyright holder for this preprint (which was not certified by peer review) is the author/funder, who has granted bioRxiv a license to display the preprint in perpetuity. It is made available under aCC-BY 4.0 International license.

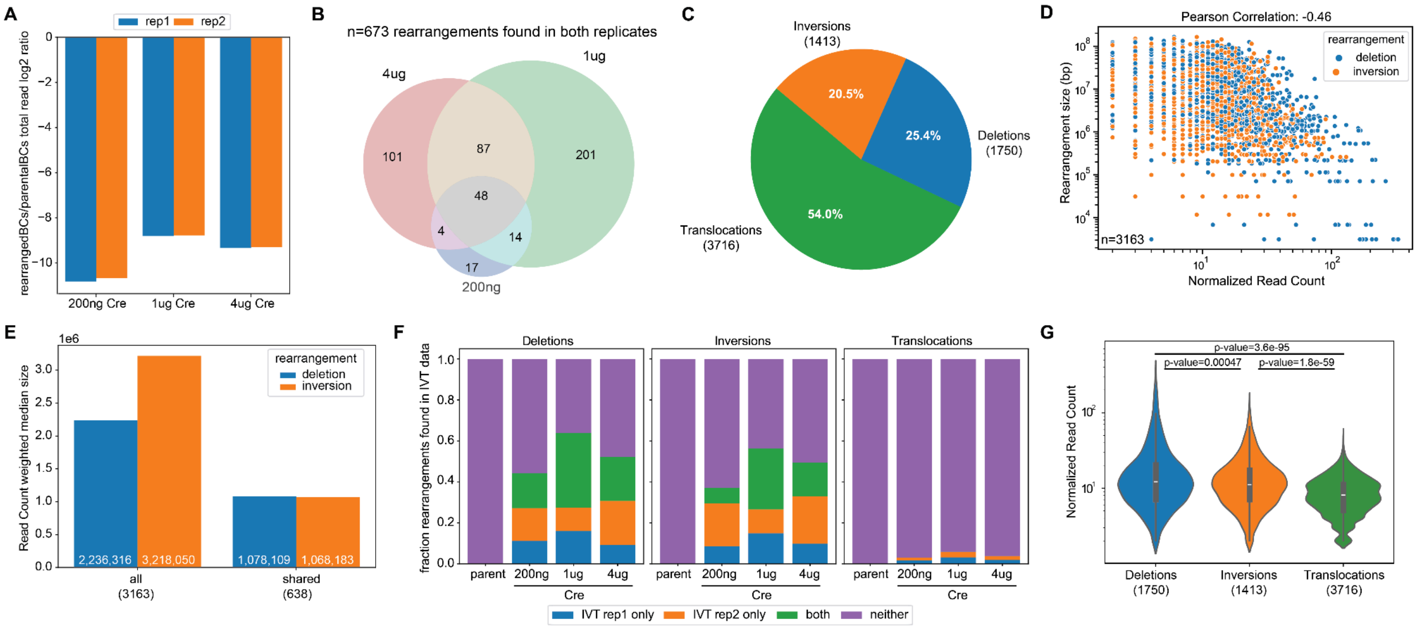

Fig S4. Characteristics of the complete set of rearrangements detected in bulk by amplicon-seq at 72h post-Cre transfection. A) Log2 ratio of total reads that contain rearranged barcode (BC) pairs to the total number of reads that contain parental BC pairs in technical replicates of each Cre transfection sample. **B)** Venn diagram depicting the overlapping relationships between Cre transfection samples for the subset of SVs that are detected in both technical replicates of each sample. **C)** Pie chart depicting the distribution of SV type for all rearrangements detected at 72h. **D)** Scatter plot of rearrangement size (*y*-axis) vs. normalized read count (*x*-axis) for deletions and inversions detected at day 3. Pearson correlation is calculated between the log10 values of the two metrics. **E)** Median size of inversions and deletions, weighted by their read count, for both the complete set of rearrangements (left) and those shared between technical replicates for a condition (right). **F)** Similar to lower part of **Fig. 3D**, the bar plot shows the proportion of each SV type (from the complete set of rearrangements at 72h) that is supported by at least one read in the IVT-seq data. **G)** Violin plots depicting the distribution of read counts for deletions, inversions and translocations for the complete set of rearrangements detected at day 3. Inset within each violin plot is a box plot of the distribution with the median value depicted as a white line, the length of the box depicting the interquartile range and the whiskers depicting the extent of the distribution. P-values are calculated using the non-parametric Mann-Whitney U test.

bioRxiv preprint doi: https://doi.org/10.1101/2024.01.22.576756; this version posted February 12, 2024. The copyright holder for this preprint (which was not certified by peer review) is the author/funder, who has granted bioRxiv a license to display the preprint in perpetuity. It is made available under aCC-BY 4.0 International license.

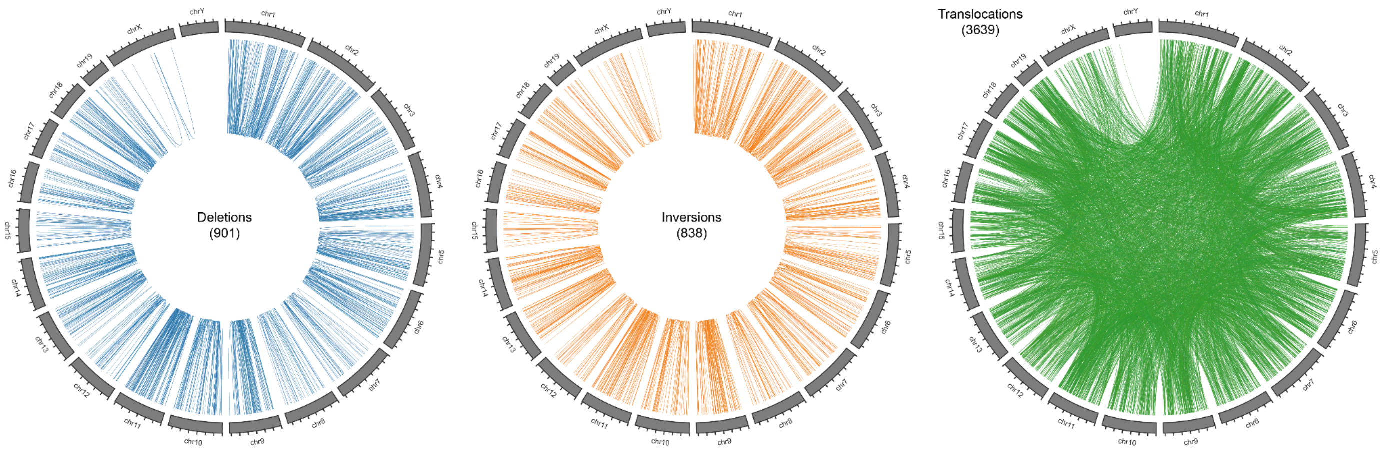

Fig S5. Circos plots of all rearrangements detected at 72h post-Cre transfection. Depicted rearrangements are from across all samples, including those that are not shared between technical replicates.

bioRxiv preprint doi: https://doi.org/10.1101/2024.01.22.576756; this version posted February 12, 2024. The copyright holder for this preprint (which was not certified by peer review) is the author/funder, who has granted bioRxiv a license to display the preprint in perpetuity. It is made available under aCC-BY 4.0 International license.

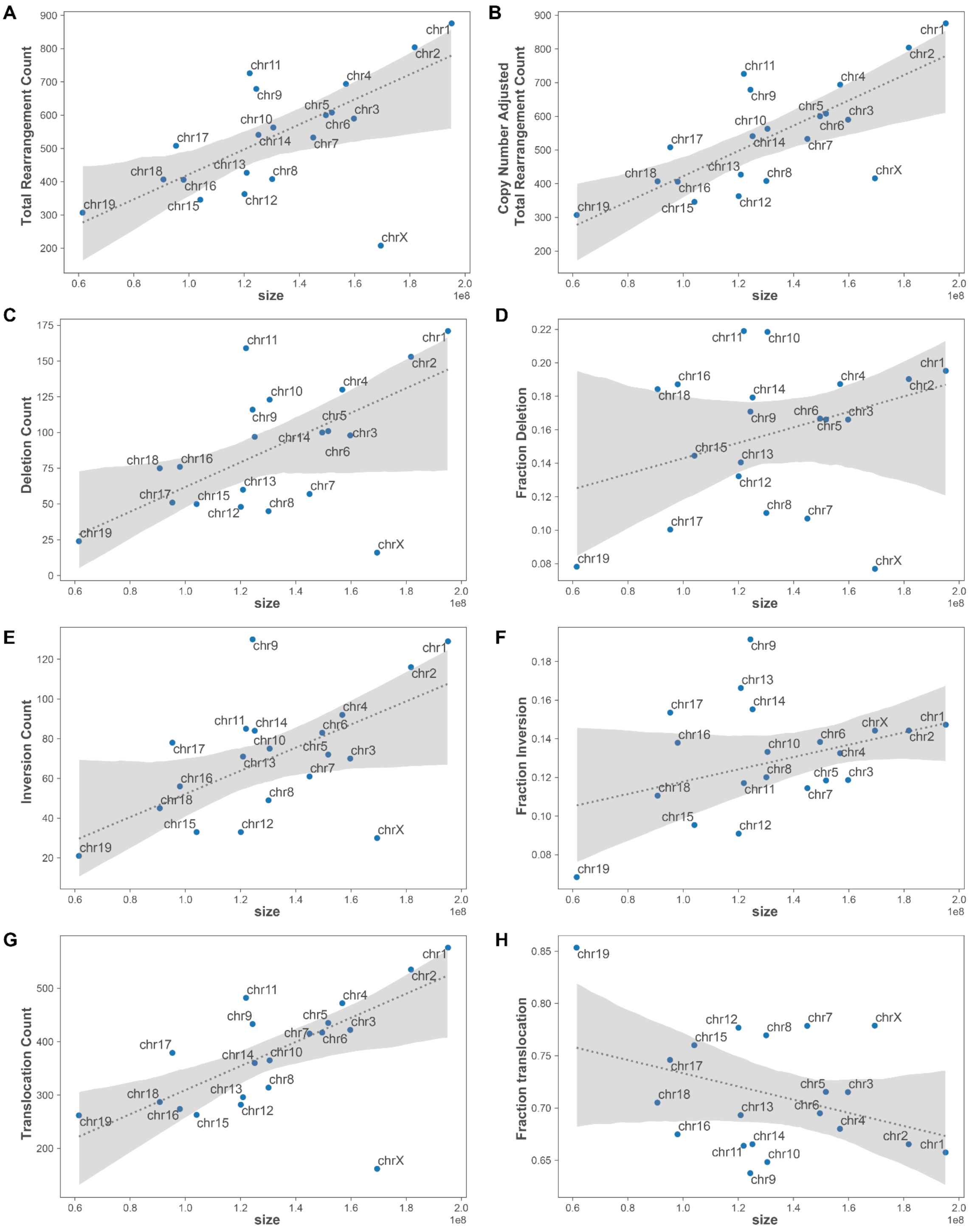

Fig S6. Distribution of number or fraction of rearrangements of each type across chromosomes. All plots in this figure depict a scatter plot of the distribution of all rearrangements detected at 72h post-Cre transfection across mouse chromosomes except chrY, with the size of the chromosomes depicted along the *x*-axis. The dotted line indicates linear regression model fit and the shaded gray areas the 95% confidence interval. The *y*-axis of each panel is either: **A)** the total number of events detected; **B)** the total number of events detected with the number of events on the X chromosome multiplied by 2 to normalize for copy number; **C)**, **E)**, **G)** the total number of deletions **(C)**, inversions **(E)** or translocations **(G)**, respectively; **D)**, **F)**, **H)** the proportion of rearrangements on a given chromosome that are deletions **(D)**, inversions **(E)** and **(G)** translocations, respectively.

bioRxiv preprint doi: https://doi.org/10.1101/2024.01.22.576756; this version posted February 12, 2024. The copyright holder for this preprint (which was not certified by peer review) is the author/funder, who has granted bioRxiv a license to display the preprint in perpetuity. It is made available under aCC-BY 4.0 International license.

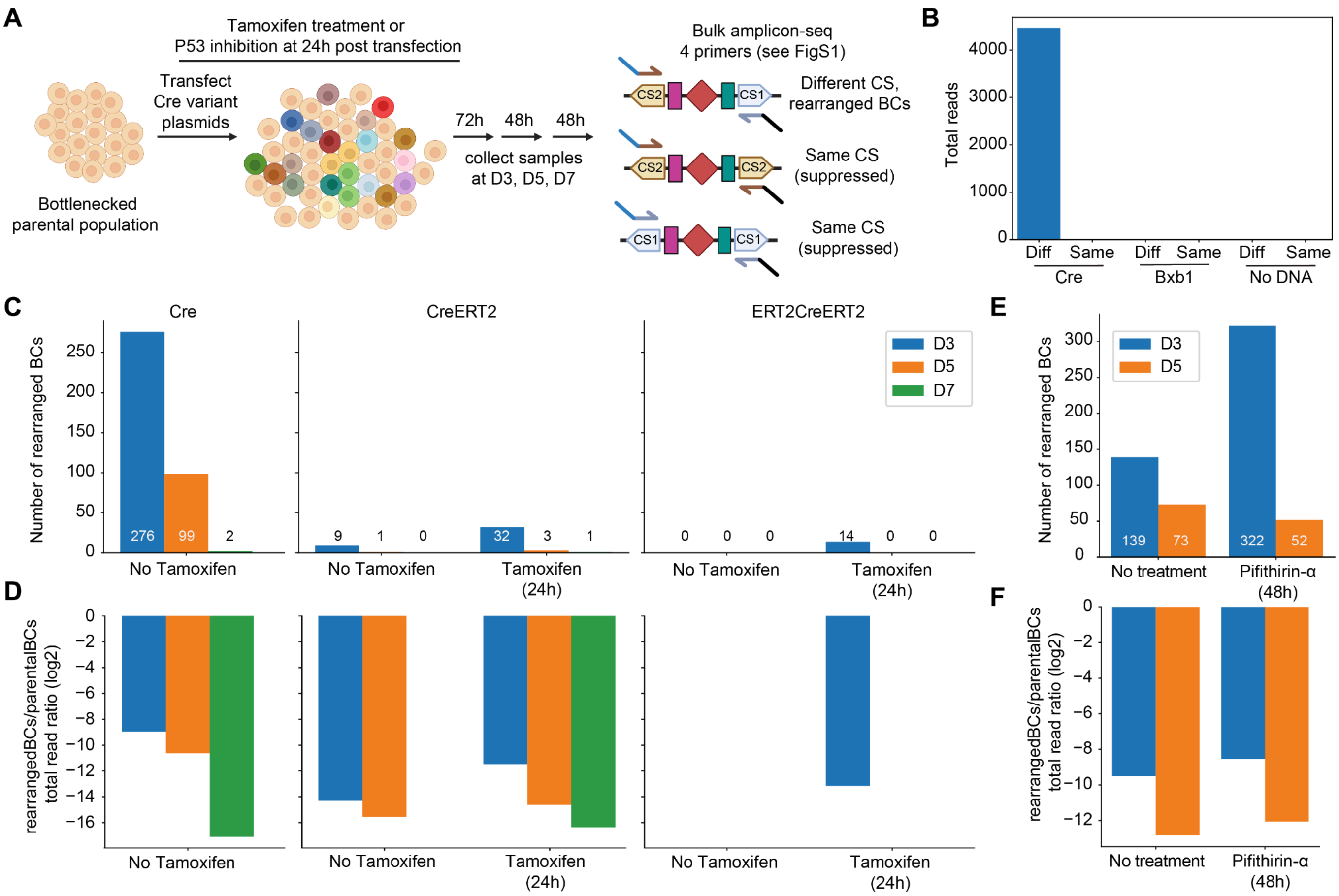

Fig S7. Rearrangements are not stably maintained in the post-Cre induction cell population and cannot be rescued by inducible Cre variants nor by p53 inhibition. A) Schematic of the long-term culture experiments with Cre variants or p53 inhibition. The possible products from the 4 primer amplicon-seq strategy (also see **Fig. S1**) are depicted to the right. **B)** Total number of reads from 4 primer amplicon-seq data generated from Cre, Bxb1 or No DNA transfected cells that contain rearranged barcode pairs. Bars are split based on whether the reads contain the same or different (diff) capture sequence on the same molecule. **C)** Number of rearranged barcode (BC) combinations detected at day 3, 5 or 7 post transfection with Cre, CreERT2 or ERT2CreERT2. Cells were either untreated or treated with tamoxifen (0.5μM) for 24 hours. **D)** Log2 ratio of total reads with rearranged BC combinations to parental BC combinations in each sample. **E)** Similar to panel **C**, the number of rearranged BCs detected at day 3 or 5 for Cre-transfected cells with or without p53 inhibitor (Pifithrin-α, 20μM). **F)** Similar to panel **D**, Log2 ratio of total reads with rearranged BC combinations to parental BC combinations for samples with or without p53 inhibitor (Pifithrin-α, 20μM). Data presented in this figure is from one replicate.

bioRxiv preprint doi: https://doi.org/10.1101/2024.01.22.576756; this version posted February 12, 2024. The copyright holder for this preprint (which was not certified by peer review) is the author/funder, who has granted bioRxiv a license to display the preprint in perpetuity. It is made available under aCC-BY 4.0 International license.

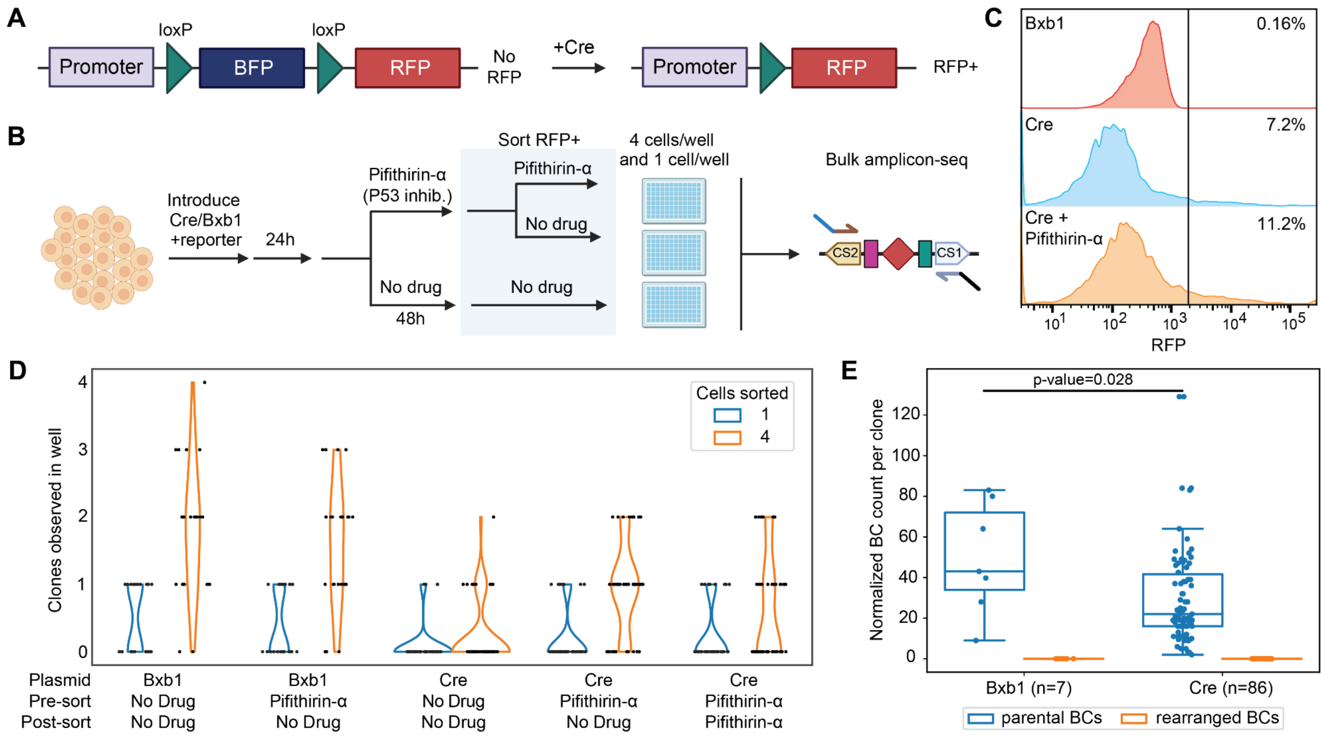

Fig S8. Single-cell sorting does not yield long-lived clones with rearrangements. A) The reporter used for this experiment encodes a floxed blue fluorescent protein (BFP) gene which is excised in the presence of Cre to constitutively express a red fluorescent protein (RFP), thus serving as a marker for Cre activity. **B)** Schematic of the single-cell sorting experiment. Cells were transfected with either Cre or Bxb1 recombinase and optionally treated with the P53 inhibitor Pifithrin-α for 48 hours before sorting out either 4 or 1 RFP positive cell(s) into single wells of 96 well plates. Genomic DNA was extracted from clones and barcodes they contained were detected using bulk amplicon-seq. **C)** Flow cytometry traces of cell populations transfected and treated as indicated. The percentage in each panel reflects the RFP positive proportion of the population. **D)** Violin plots depicting the number of clones observed by eye per well 7 days after sorting, separated by the number of cells initially sorted into that well. **E)** Boxplots of the number of parental or rearranged barcode combinations (BCs) observed per well, normalized for the number of clones that were observed in that well. The horizontal solid line indicates the median, the length of the box depicts the interquartile range and the whiskers depict the extent of the distribution minus outliers. Data presented in this figure is from one replicate.

bioRxiv preprint doi: https://doi.org/10.1101/2024.01.22.576756; this version posted February 12, 2024. The copyright holder for this preprint (which was not certified by peer review) is the author/funder, who has granted bioRxiv a license to display the preprint in perpetuity. It is made available under aCC-BY 4.0 International license.

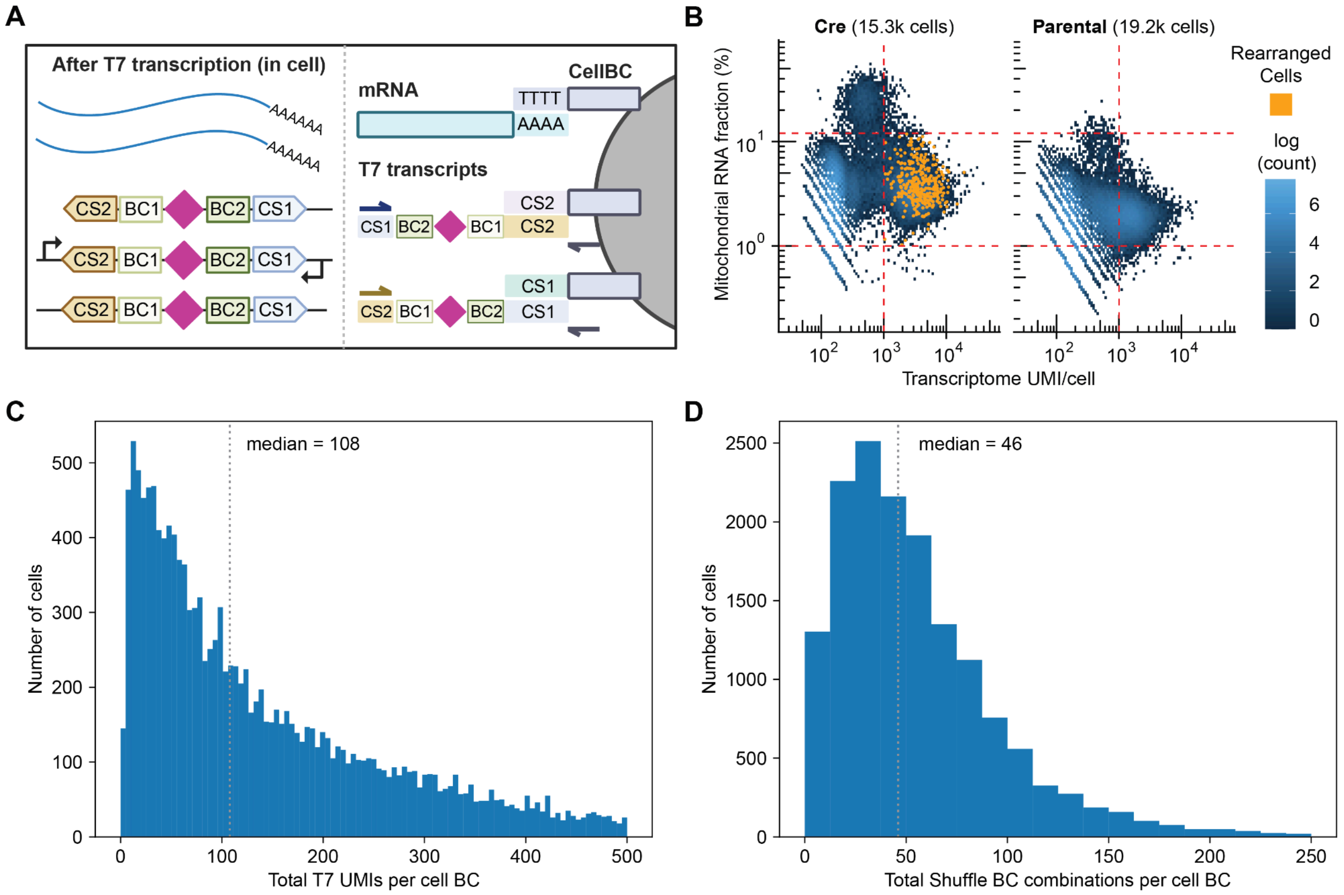

Fig S9. Detection of shuffle cassette barcodes in scRNA-seq data. A) After fixation and IVT with T7 polymerase, cells contain both mRNA from endogenous genes as well as RNA from shuffle cassettes. Both of these RNA species can be captured using 10X Genomics gel beads that contain complementary sequences for the polyA on mRNA and capture sequence 1 and 2 (CS1, CS2) found on the T7 derived shuffle transcripts. **B)** Scatter plots of mitochondrial RNA fraction vs. transcriptome unique molecular identifier (UMI) counts per cell detected in the Cre and parental conditions. All cells associated with a rearranged barcode pair at >1 UMI (n=320) are colored yellow. **C)** Histogram of total T7 derived UMIs per cell barcode (BC) with the median value represented by a vertical dotted gray line. **D)** Histogram of total shuffle BC combinations detected per cell in T7 transcripts. Median value is again depicted by a vertical dotted gray line.

bioRxiv preprint doi: https://doi.org/10.1101/2024.01.22.576756; this version posted February 12, 2024. The copyright holder for this preprint (which was not certified by peer review) is the author/funder, who has granted bioRxiv a license to display the preprint in perpetuity. It is made available under aCC-BY 4.0 International license.

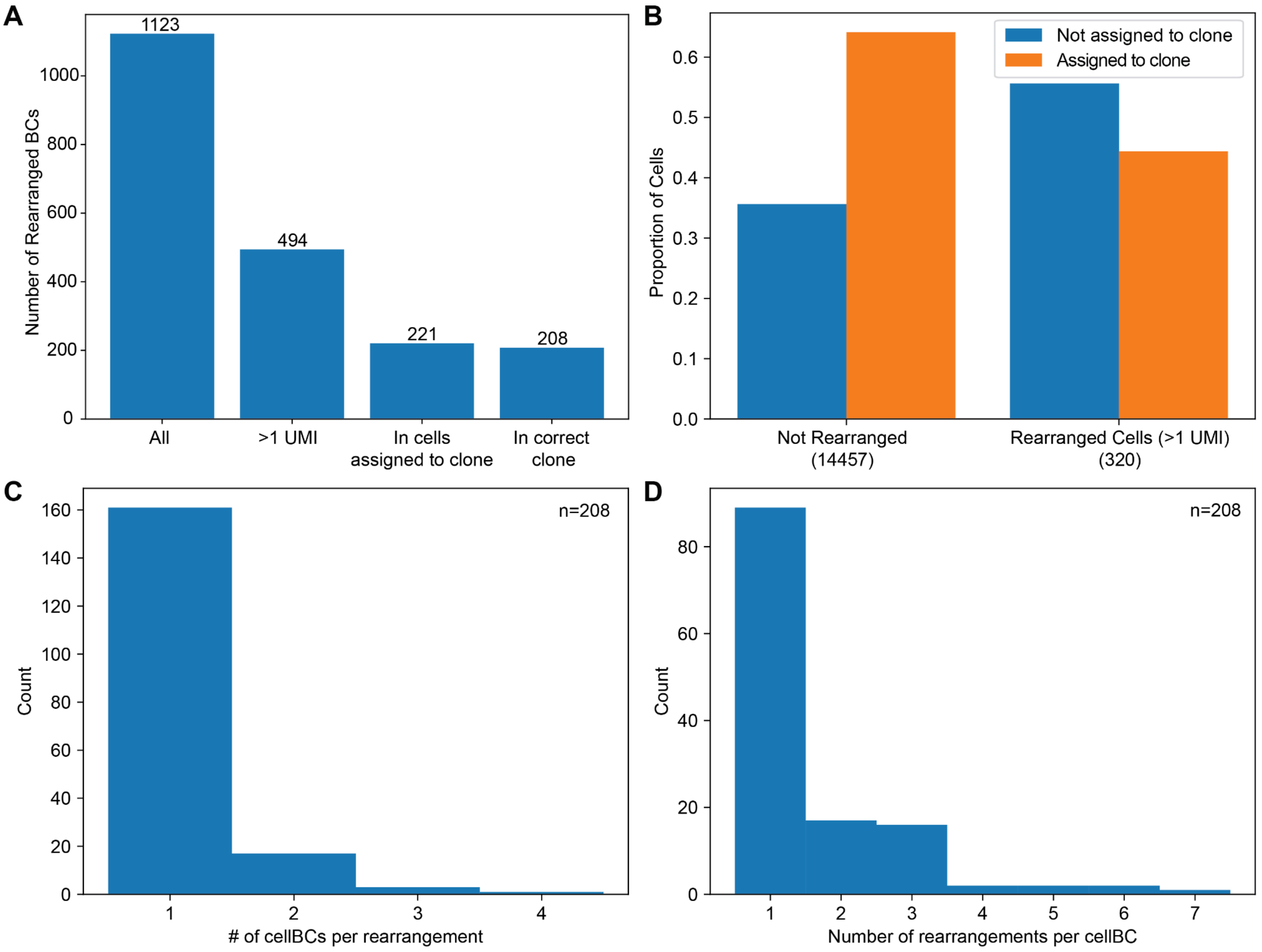

Fig S10. Characteristics of T7-derived barcodes detected in scRNA-seq data. A) Number of rearranged BC combinations that are detected in the single cell Cre dataset at successive stages of filtering: all cells, rearrangements detected at >1 UMI, rearrangements at >1 UMI in cells that could be assigned to a clonotype and lastly, rearrangements for which the identity of the rearranged BC pair was congruent with the clonotype assignment. That is, both BCs were detected in the same parental clone. **B)** The proportion of cells that can be assigned to a parental clone for cells that contain a rearrangement or do not. Clonotype assignment is determined by the set of T7 barcodes (BCs) within them, detected with >1 unique molecular identifier (UMI). Cells were considered assigned to a clone if at least 75% of the T7 BCs detected in that cell at >1 UMI belong to that specific clone.**C)** Histogram depicting the number of unique cell barcodes associated with a particular rearrangement. **D)** Histogram depicting the number of rearranged barcodes per cell.

bioRxiv preprint doi: https://doi.org/10.1101/2024.01.22.576756; this version posted February 12, 2024. The copyright holder for this preprint (which was not certified by peer review) is the author/funder, who has granted bioRxiv a license to display the preprint in perpetuity. It is made available under aCC-BY 4.0 International license.

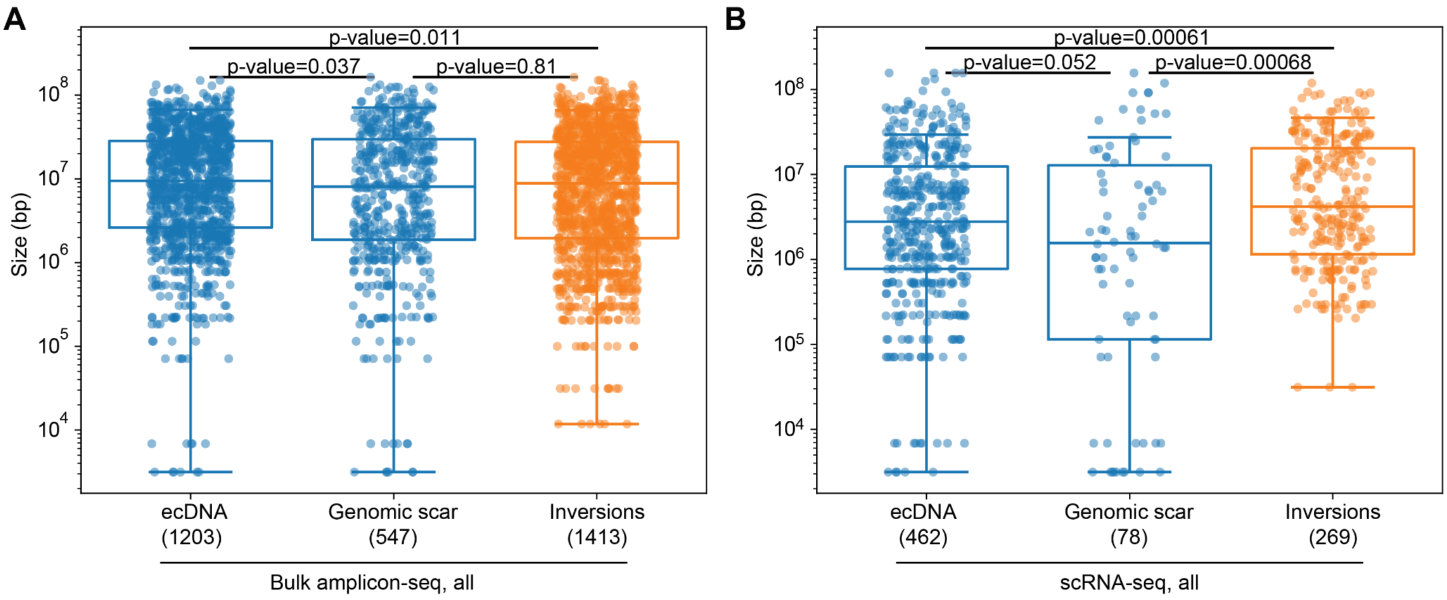

Fig S11. Size distribution of ecDNAs relative to genomic deletions and inversions. A) and **B)** Size of each ecDNA, deletion scar and inversion event detected in bulk amplicon-seq data (all rearrangements) and scRNA-seq (all rearrangements) respectively. Depicted p-value is calculated using the non-parametric Mann-Whitney U test. The horizontal solid line indicates the median, the length of the box depicts the interquartile range and the whiskers depict the extent of the distribution minus outliers

bioRxiv preprint doi: https://doi.org/10.1101/2024.01.22.576756; this version posted February 12, 2024. The copyright holder for this preprint (which was not certified by peer review) is the author/funder, who has granted bioRxiv a license to display the preprint in perpetuity. It is made available under aCC-BY 4.0 International license.

Supplementary Movie 1. Circos plots for the 129 rearranged single-cells detected in the dataset. Thickness of the line depicting each rearrangement is proportional to its unique molecular identifier (UMI) count in that cell.

@ 10x_v2.mp4
